# Supplementary material for: Medical staff’s perspectives on patients’ anxieties and interventions in a rehabilitation ward: A qualitative study
Source: PLoS One. 2025 Aug 7;20(8):e0329443. doi: 10.1371/journal.pone.0329443 (PMC12331052; doi:10.1371/journal.pone.0329443)
Supplement: S1 Fig — Cluster 1, prognosis of physical function; Cluster 2, differences from the acute care ward; Cluster 3, prospects of social life; Cluster 4, prognosis of cognitive disorders; Cluster 5, prospects for rehabilitation plans; and Cluster 6, family situation. Dotted vertical line: Threshold of the agglomeration dissimilarity coefficient. (DOCX) [file pone.0329443.s001.docx]

**S1 Fig.** Cluster dendrogram of the patients’ anxieties in the early phase of hospitalization

Cluster 1, prognosis of physical function; Cluster 2, differences from the acute care hospital; Cluster 3, prospects of social life; Cluster 4, prognosis of cognitive disorders; Cluster 5, prospects for rehabilitation plans; and Cluster 6, family situation. Dotted vertical line: Threshold of the agglomeration dissimilarity coefficient.
