# Supplementary material for: Medical staff’s perspectives on patients’ anxieties and interventions in a rehabilitation ward: A qualitative study
Source: PLoS One. 2025 Aug 7;20(8):e0329443. doi: 10.1371/journal.pone.0329443 (PMC12331052; doi:10.1371/journal.pone.0329443)
Supplement: S3 Fig — Cluster 1, lack of information about the medical follow-up system after discharge; Cluster 2, decline in physical function after discharge; Cluster 3, differences in the physical and interpersonal environments between the hospital and home; and Cluster 4, role reacquisition (return to work, household chores). Dotted vertical line: Threshold of the agglomeration dissimilarity coefficient. (DOCX) [file pone.0329443.s003.docx]

**S3 Fig.** Cluster dendrogram of the patients’ anxieties in the late phase of hospitalization

Cluster 1, lack of information about the medical follow-up system after discharge; Cluster 2, decline in physical function after discharge; Cluster 3, differences in the physical and interpersonal environments between the hospital and home; and Cluster 4, role reacquisition (return to work, household chores). Dotted vertical line: Threshold of the agglomeration dissimilarity coefficient.
