# Supplementary material for: Medical staff’s perspectives on patients’ anxieties and interventions in a rehabilitation ward: A qualitative study
Source: PLoS One. 2025 Aug 7;20(8):e0329443. doi: 10.1371/journal.pone.0329443 (PMC12331052; doi:10.1371/journal.pone.0329443)
Supplement: S4 Fig — Cluster 1, explanation of the current situation by medical staff; Cluster 2, setting and sharing goals; Cluster 3, support for family and patient interactions; and Cluster 4, explanation of the rehabilitation treatment plan by physiatrists. Dotted vertical line: Threshold of the agglomeration dissimilarity coefficient. (DOCX) [file pone.0329443.s004.docx]

**S4 Fig.** Cluster dendrogram of the interventions for patients’ anxieties in the early phase of hospitalization.

Cluster 1, explanation of the current situation by medical staff; Cluster 2, setting and sharing goals; Cluster 3, support for family and patient interactions; and Cluster 4, explanation of the rehabilitation treatment plan by physiatrists. Dotted vertical line: Threshold of the agglomeration dissimilarity coefficient.
