# Supplementary material for: Medical staff’s perspectives on patients’ anxieties and interventions in a rehabilitation ward: A qualitative study
Source: PLoS One. 2025 Aug 7;20(8):e0329443. doi: 10.1371/journal.pone.0329443 (PMC12331052; doi:10.1371/journal.pone.0329443)
Supplement: S5 Fig — Cluster 1, sharing information with family members; Cluster 2, setting and sharing goals; Cluster 3, assistance in coordinating return to work; Cluster 4, feedback using numerical data; Cluster 5, feedback about improvement in activities of daily living; Cluster 6, feedback using videos; and Cluster 7, explanation of the rehabilitation treatment plan by a physiatrist. Dotted vertical line: Threshold of the agglomeration dissimilarity coefficient. (DOCX) [file pone.0329443.s005.docx]

**S5 Fig.** Cluster dendrogram of the interventions for patients’ anxieties in the middle phase of hospitalization

Cluster 1, sharing information with family members; Cluster 2, setting and sharing goals; Cluster 3, assistance in coordinating return to work; Cluster 4, feedback using numerical data; Cluster 5, feedback about improvement in activities of daily living; Cluster 6, feedback using videos; and Cluster 7, explanation of the rehabilitation treatment plan by a physiatrist. Dotted vertical line: Threshold of the agglomeration dissimilarity coefficient.
