# Supplementary material for: Medical staff’s perspectives on patients’ anxieties and interventions in a rehabilitation ward: A qualitative study
Source: PLoS One. 2025 Aug 7;20(8):e0329443. doi: 10.1371/journal.pone.0329443 (PMC12331052; doi:10.1371/journal.pone.0329443)
Supplement: S6 Fig — Cluster 1, simulation of movements required after discharge; Cluster 2, providing information about care services; Cluster 3, providing information for outpatient visits; Cluster 4, guidance for self-exercises; Cluster 5, home visit and investigation; and Cluster 6, guidance of movements procedures and assistance methods. Dotted vertical line: Threshold of the agglomeration dissimilarity coefficient. (DOCX) [file pone.0329443.s006.docx]

**S6 Fig.** Cluster dendrogram of interventions for patients’ anxieties in the late phase of hospitalization

Cluster 1, simulation of movements required after discharge; Cluster 2, providing information about care services; Cluster 3, providing information for outpatient visits; Cluster 4, guidance for self-exercises; Cluster 5, home visit and investigation; and Cluster 6, guidance of movements procedures and assistance methods. Dotted vertical line: Threshold of the agglomeration dissimilarity coefficient.
