# Supplementary material for: Medical staff’s perspectives on patients’ anxieties and interventions in a rehabilitation ward: A qualitative study
Source: PLoS One. 2025 Aug 7;20(8):e0329443. doi: 10.1371/journal.pone.0329443 (PMC12331052; doi:10.1371/journal.pone.0329443)
Supplement: S1 Table — (DOCX) [file pone.0329443.s007.docx]

**S1 Table.** Correlations between patients’ anxiety and the types of interventions in the early phase of hospitalization

|  | | Types of interventions | | | |
| --- | --- | --- | --- | --- | --- |
|  |  | Explanation of the current situation by medical staff | Explanation of the rehabilitation treatment plan by physiatrists | Setting and sharing goals | Support for family and patient interactions |
| Patients’  anxieties | Prospects for rehabilitation plans | ✔ | ✔ | ✔ |  |
|  | Prognosis of physical function |  | ✔ | ✔ |  |
|  | Prognosis of cognitive disorders | ✔ | ✔ | ✔ |  |
|  | Family situation |  |  |  | ✔ |
|  | Differences from the acute care ward | ✔ | ✔ |  |  |
|  | Prospects of social life | ✔ | ✔ |  |  |
