# Supplementary material for: Medical staff’s perspectives on patients’ anxieties and interventions in a rehabilitation ward: A qualitative study
Source: PLoS One. 2025 Aug 7;20(8):e0329443. doi: 10.1371/journal.pone.0329443 (PMC12331052; doi:10.1371/journal.pone.0329443)
Supplement: S2 Table — (DOCX) [file pone.0329443.s008.docx]

**S2 Table.** Correlations between patients’ anxiety and the types of interventions in the middle phase of hospitalization

|  | | Types of interventions | | | | | | |
| --- | --- | --- | --- | --- | --- | --- | --- | --- |
|  |  | Explanation of the rehabilitation treatment plan by physiatrists | Setting and sharing goals | Sharing information with family members | Feedback about improvement in ADL | Feedback using numerical data | Feedback using videos | Assistance in coordinating return to work |
| Patients’  anxieties | Prospects for rehabilitation plans | ✔ | ✔ |  |  |  |  |  |
|  | Lack of feelings of improvement |  |  |  | ✔ | ✔ | ✔ |  |
|  | Prognosis of physical function | ✔ | ✔ |  | ✔ | ✔ | ✔ |  |
|  | Prospects of social life | ✔ | ✔ | ✔ |  |  |  | ✔ |

ADL = activities of daily living
