# Supplementary material for: Medical staff’s perspectives on patients’ anxieties and interventions in a rehabilitation ward: A qualitative study
Source: PLoS One. 2025 Aug 7;20(8):e0329443. doi: 10.1371/journal.pone.0329443 (PMC12331052; doi:10.1371/journal.pone.0329443)
Supplement: S3 Table — (DOCX) [file pone.0329443.s009.docx]

**S3 Table.** Correlations between patients’ anxiety and the types of interventions in the late phase of hospitalization

|  | | Types of interventions | | | | | |
| --- | --- | --- | --- | --- | --- | --- | --- |
|  |  | Providing information for outpatient visits | Guidance of movements procedures and assistance methods | Providing information about nursing care services | Simulation of movements required after discharge | Home visit and investigation | Guidance for self-exercises |
| Patients’  anxieties | Decline in physical function after discharge |  |  |  |  |  | ✔ |
|  | Role reacquisition (return to work, household chores) |  |  | ✔ | ✔ | ✔ |  |
|  | Lack of information about the medical follow-up system after discharge | ✔ |  | ✔ |  |  |  |
|  | Differences in the physical and interpersonal environments between the hospital and home |  | ✔ |  | ✔ | ✔ |  |
