# Supplementary material for: Medical staff’s perspectives on patients’ anxieties and interventions in a rehabilitation ward: A qualitative study
Source: PLoS One. 2025 Aug 7;20(8):e0329443. doi: 10.1371/journal.pone.0329443 (PMC12331052; doi:10.1371/journal.pone.0329443)
Supplement: S1 File — (DOCX) [file pone.0329443.s010.docx]

| Interview Topic Guide |
| --- |
| At the beginning of each interview, the interviewer verbally recorded the date and participant ID. Example: “December 2nd, participant ID number 02”   1. **Anxieties in the Early Phase of Hospitalization**   What are the anxieties in patients during the early phase of hospitalization in a convalescent rehabilitation ward?  **Prompts**  ・Please consider differences depending on age, gender, physical condition, and activity level.  ・The early phase refers to the first 1–2 weeks after admission, following initial testing and administrative procedures, and after rehabilitation has started.  ・Examples of anxiety areas: body function, home situation, work, hospital life.   1. **Intervention in the Early Phase of Hospitalization**   How do you address those anxieties in the early phase?  **Prompt**  ・Providing detailed explanations about therapy, discussing rehabilitation goals with the patient, offering general information about the illness.   1. **Anxieties in the Middle Phase of Hospitalization**   What are the anxieties in patients during the middle phase of hospitalization in a convalescent rehabilitation ward?  **Prompts**  ・This refers to the period after initial assessments have been completed and rehabilitation is well underway, but before the discharge date is determined.  ・Examples of anxiety areas: body function, home situation, work, hospital life.   1. **Intervention in the Early Phase of Hospitalization**   How do you address those anxieties in the middle phase?  **Prompt**  ・Providing detailed explanations about therapy, discussing rehabilitation goals with the patient, offering general information about the illness.   1. **Anxieties in the Late Phase of Hospitalization**   What are the anxieties in patients during the late phase of hospitalization in a convalescent rehabilitation ward?  **Prompts**  ・This refers to the period after the discharge date has been decided, typically several weeks before discharge.  ・Examples of anxiety areas: body function, home situation, work, hospital life.   1. **Intervention in the Late Phase of Hospitalization**   How do you address those anxieties in the late phase?  **Prompt**  ・Providing detailed explanations about therapy, discussing rehabilitation goals with the patient, offering general information about the illness. |

| **インタビューガイド** |
| --- |
| インタビュー開始時：日付と管理番号をインタビュー実施者が読み上げてから開始して下さい．  例：「12月2日　管理番号02です．」   1. **患者が入院期間の前期でよく感じる不安はどのようなものだと思いますか？**   　回答の促し例：年齢差，男女差，機能・活動・  　入院期間の前半とは，一通りの入院時の検査や手続きが終了し，リハビリが始まってから1-2週目あたりです  患者が入院してリハビリが始まって比較的早い段階でよく経験する不安事項とはなんですか？  　例えば，体のことや家庭生活，仕事，入院生活などではどうでしょうか？   1. **その（入院前期の）不安に対して，どのように対応していますか？**   　回答の促し例：  　例えば，訓練内容について詳しく説明するとか，目標について患者と話し合うとか，疾患についての一般情報を提供するなどはどうでしょうか？   1. **患者が入院期間の中期でよく感じる不安はどのようなものだと思いますか？**   　回答の促し例：  入院期間の中期とは，入院時の検査が終わり，リハビリが始まってから具体的な退院日が決まるまでの期間です．  　例えば，体のことや家庭生活，仕事，入院生活などではどうでしょうか？   1. **その（入院中期の）不安に対して，どのように対応していますか？**   　回答の促し例：  　例えば，訓練内容について詳しく説明するとか，目標について患者と話し合うとか，疾患についての一般情報を提供するなどはどうでしょうか？   1. **患者が入院期間の後期でよく感じる不安はどのようなものだと思いますか？**   　回答の促し例：  入院期間の後半とは，具体的な退院の日程が決まる時期，退院まで残り数週間の時期です  患者が，入院の後半，退院が決まりそうな段階でよく経験する不安事項とはなんですか？  　例えば，体のことや家庭生活，仕事，入院生活などではどうでしょうか？   1. **その（入院後期の）不安に対して，どのように対応していますか？**   　回答の促し例：  　例えば，訓練内容について詳しく説明するとか，目標について患者と話し合うとか，疾患についての一般情報を提供するなどはどうでしょうか？ |
