# Supplementary material for: Medical staff’s perspectives on patients’ anxieties and interventions in a rehabilitation ward: A qualitative study
Source: PLoS One. 2025 Aug 7;20(8):e0329443. doi: 10.1371/journal.pone.0329443 (PMC12331052; doi:10.1371/journal.pone.0329443)
Supplement: Attachment — Submitted filename: comments.docx [file pone.0329443.s012.docx]

**General Comments**

The manuscript is methodologically sound, clearly structured, and provides useful insights into how rehabilitation staff perceive and address the emotional and psychological challenges of patients. However, a few areas require clarification, and some improvements could strengthen the manuscript’s rigor and practical relevance.

**Major Comments**

**Abstract**

Well-structured, but it would benefit from reporting specific examples of anxieties and interventions to provide more concrete insight.

**Introduction**

Consider adding a brief literature comparison with other qualitative studies addressing similar topics in different cultural or clinical settings.

**Methods**

Clarify if interview transcripts were returned to participants for review (member checking).

While the rationale for not interviewing patients (concerns about recall bias and individual variability) is mentioned, it still raises a limitation in terms of representativeness of patient voices. Include a more detailed justification for the exclusive use of medical staff input and discuss whether a triangulation approach involving both patient and staff perspectives might offer a richer analysis in future work.

**Results**

The current clustering provides breadth but lacks deep thematic interpretation. Complement the cluster results with qualitative excerpts or mini-case examples from interviews to give depth and voice to the findings.

**Discussion**

The implications for practice could be expanded. For example:

How can staff training be improved using these insights? Could an intervention toolkit or checklist be developed from these findings?

Author could write in implication section below phrase to strengthen the study findings.

“Recent evidence has demonstrated that psychological distress and suicide attempts follow distinct temporal patterns, with increased risk during afternoon and evening hours, particularly among vulnerable populations such as young and married individuals [Ref:sheikh et al. doi; https://doi.org/10.1016/j.bbii.2024.100072]. Our findings highlight the need for timely psychosocial interventions that are sensitive to daily fluctuations in mood and anxiety, especially in rehabilitation settings where patients face uncertainties about recovery and discharge.”

The study involved 17 staff members from a single hospital, potentially limiting generalizability.

Acknowledge this more clearly as a limitation and discuss if saturation was reached in the interviews. Consider explaining if there was any variation in responses between different professional roles (OT, PT, nurse, etc.).
